# CCR4 is a determinant of melanoma brain metastasis

**DOI:** 10.18632/oncotarget.16076

**Published:** 2017-03-10

**Authors:** Anat Klein, Orit Sagi-Assif, Tsipi Meshel, Alona Telerman, Sivan Izraely, Shlomit Ben-Menachem, Jagadeesh Bayry, Diego M. Marzese, Shuichi Ohe, Dave S.B. Hoon, Neta Erez, Isaac P. Witz

**Affiliations:** ^1^ Department of Cell Research and Immunology, George S. Wise Faculty of Life Sciences, Tel-Aviv University, Tel Aviv, Israel; ^2^ Inserm Unité 1138, Center de Recherche des Cordeliers, Université Pierre et Marie Curie, Université, Paris Descartes, Paris, France; ^3^ Department of Molecular Oncology, John Wayne Cancer Institute at Providence Saint John's Health Center, Santa Monica, CA, USA; ^4^ Department of Pathology, Sackler School of Medicine, Tel Aviv University, Tel Aviv, Israel

**Keywords:** CCR4, CCL17, melanoma, brain, metastasis

## Abstract

We previously identified the chemokine receptor CCR4 as part of the molecular signature of melanoma brain metastasis. The aim of this study was to determine the functional significance of CCR4 in melanoma brain metastasis. We show that CCR4 is more highly expressed by brain metastasizing melanoma cells than by local cutaneous cells from the same melanoma. Moreover, we found that the expression of CCR4 is significantly higher in paired clinical specimens of melanoma metastases than in samples of primary tumors from the same patients. Notably, the expression of the CCR4 ligands, *Ccl22* and *Ccl17* is upregulated at the earliest stages of brain metastasis, and precedes the infiltration of melanoma cells to the brain. *In-vitro*, CCL17 induced migration and transendothelial migration of melanoma cells. Functionally, human melanoma cells over-expressing CCR4 were more tumorigenic and produced a higher load of spontaneous brain micrometastasis than control cells. Blocking CCR4 with a small molecule CCR4 antagonist *in-vivo*, reduced the tumorigenicity and micrometastasis formation of melanoma cells. Taken together, these findings implicate CCR4 as a driver of melanoma brain metastasis.

## INTRODUCTION

It is well established that interactions between tumor cells with non-tumor cells in the tumor microenvironment drive tumor progression towards metastasis [1–3]. These tumor-microenvironment interactions are bidirectional and each interaction partner regulates and shapes the phenotype of the other partner.

Determinants of metastasis are cells or molecules whose activity is required for the targeted migration of metastasizing tumor cells to the secondary target organ and for their survival and propagation within this organ. Both intrinsic tumor factors as well as microenvironmental factors could function as determinants of metastasis and both are target candidates for novel therapies.

To identify and characterize determinants of metastasis we developed human to mouse xenograft models comprising non-metastatic and metastatic variants originating in the same human neuroblastoma or melanoma [4–6] tumors. Since these variants have identical genetic backgrounds, transcriptomic, proteomic, genomic and epigenomic differences between non-metastatic and metastatic variants can be attributed to differences between their metastatic phenotypes. Employing melanoma variants, we established a molecular signature of melanoma brain metastasis (MBM) including among others, the chemokine receptor CCR4 [5, 6] and the tight junction protein Claudin-1 [7].

The chemokine (C-C) motif receptor 4 (CCR4) and its ligands CCL17 and CCL22 are regulators of immune responses especially those mediated by regulatory T cells (Tregs) and TH2 cells [8, 9]. In ovarian cancer CCR4^+^Tregs migrate towards CCL22 produced by tumor cells or by the tumor microenvironment, thereby creating favorable conditions for tumor growth. High levels of Treg cells were detected in a variety of solid tumors such as breast, colorectal, esophageal, gastric, lung and liver cancer and melanoma [10]. CCR4 is associated with high tumor recurrence and poor prognosis of gastric cancer patients [11].

Cutaneous and brain-metastasizing melanoma variants stimulated with the CCR4 ligand CCL22, showed a differential AKT phosphorylation pattern [6]. The expression of this chemokine receptor was regulated by the brain microenvironment, as brain derived-soluble factors upregulated CCR4 expression in melanoma cells [12]. We also demonstrated that highly malignant vemurafenib (BRAFmt inhibitor)-resistant human melanoma cells expressed high levels of CCR4. CCL17 and CCL22 expression was upregulated in several organs including lung, liver and brain of the tumor-bearing nude mice harboring these highly malignant tumors [13].

These findings led us to investigate the functional significance of CCR4 in melanoma brain metastasis both at the clinical setting as well as in experimental models.

## RESULTS

### CCR4 is upregulated in brain-tropic melanoma cells

**Figure 1:**
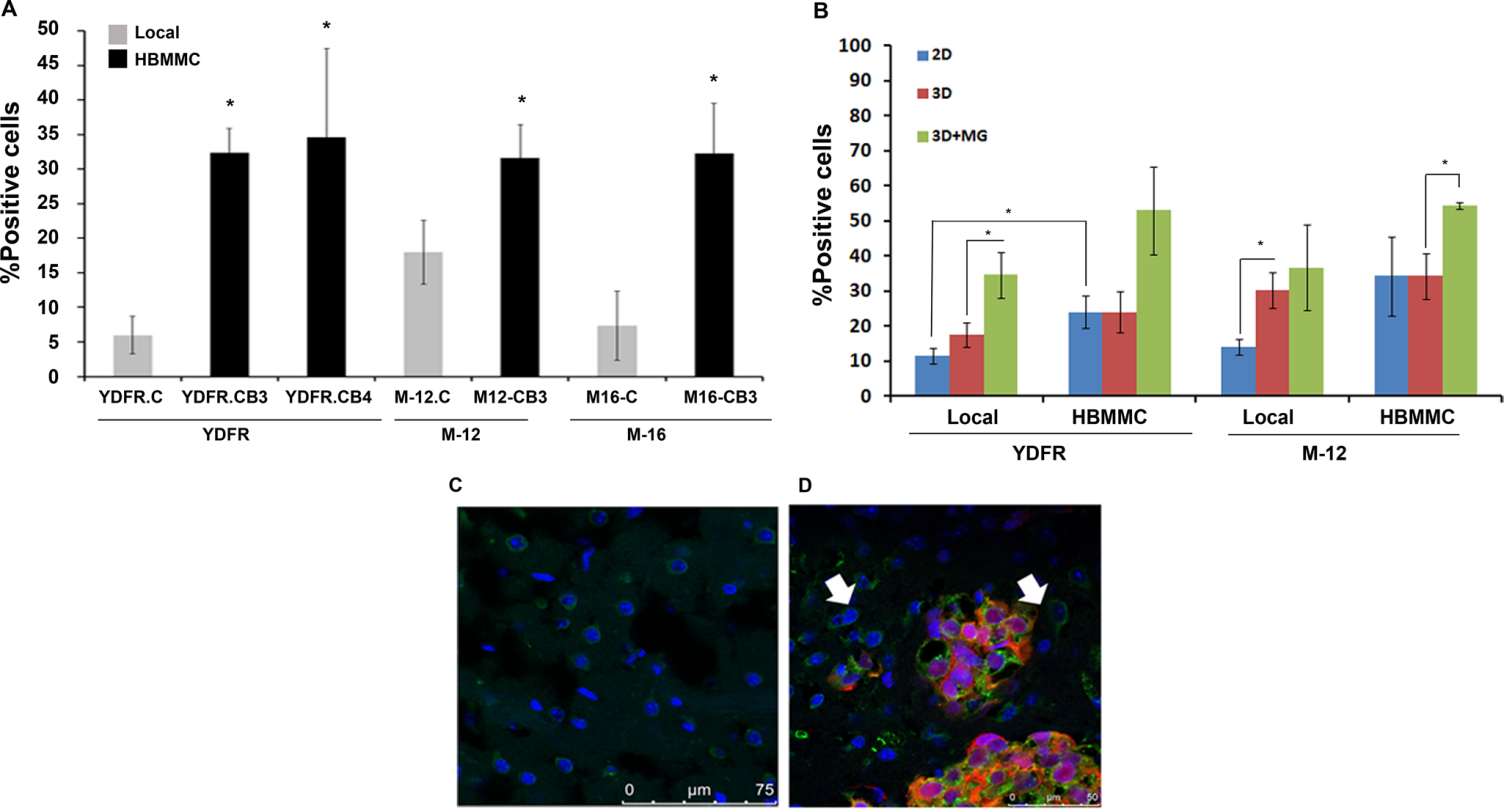
CCR4 is upregulated in brain-tropic melanoma cells (**A**) FACS analysis for CCR4 expression by local melanoma variants compared to brain metastasizing melanoma variants (HBMMC) from 3 different melanomas (YDFR, M-12, M-16). Graphs show the average % positive cells in three independent experiments, *p < 0.05. (**B**) FACS analysis for CCR4 expression of local and of brain metastasizing melanoma cells (HBMMC). Melanoma cells grown in 3D cultures (3D) express higher levels of CCR4 than cells grown in 2D cultures (2D). Addition of microglia-derived soluble factors to melanoma cells grown in 3D cultures (3D+MG) upregulated the expression of CCR4 even more. Graphs show the average % positive cells in three independent experiments, *p < 0.05. (**C**–**D**) Brain sections of normal mice (C) and of mice inoculated via the intra-cardiac route with 1 × 10^6^ mCherry-HBMMC (D). Brain sections were stained by immunofluorescence for CCR4 (green). Melanoma macrometastases are red and cell nuclei are blue (DAPI), Magnification: ×63. Scale bar = 75 μm (C), Scale bar = 50 μm (D). Arrows indicate CCR4 expressing stromal cells in the brain microenvironment.

Our previous results have indicated that the chemokine receptor CCR4 is significantly upregulated in brain-metastasizing melanoma cells [6]. To expand this observation, we evaluated the expression of CCR4 in a panel of three patient-derived cell lines of MBM (YDFR, UCLA-SO-M12, and UCLA-SO-M16). Flow cytometry analysis revealed that brain metastasizing human melanoma cell variants (YDFR.CB4, M12.CB3 and M16.CB2 - referred here after as HBMMC) [5], expressed higher levels of CCR4 than cells from the corresponding local YDFR.C, M12.C and M16.C variants (Figure 1A).

We next asked whether the difference in CCR4 expression between local and HBMMC is also manifested under three-dimensional (3D) growth conditions, which represent more closely the *in-vivo* reality [14–16]. We found that CCR4 expression is significantly higher (*p* < 0.05) on local melanoma variants propagating in 3D culture than on the same cells growing under 2D conditions (Figure 1B), suggesting that the extracellular matrix in 3D cultures has a regulatory effect on the expression of CCR4.

As mentioned above, the expression of CCR4 is regulated by the brain microenvironment [12]. In an effort to create an *in-vitro* system mimicking the brain microenvironment, we added soluble factors derived from microglia cells, an important constituent of the brain microenvironment, to cutaneous and HBMMC grown in 3D culture. The results (Figure 1B) demonstrated that microglia-derived soluble factors upregulated the expression of CCR4 by melanoma cells.

The next set of experiments was aimed to establish whether CCR4 is expressed by brain-metastasizing melanoma cells *in-vivo*. The results presented in Figure 1C and 1D show that CCR4 is highly expressed by human melanoma cells in brain metastatic lesions of the xenografted nude mice.

Taken together, these results show that the brain microenvironment is involved in the regulation of CCR4 expression by MBM.

### CCR4 expression is highly upregulated in clinical samples of melanoma brain metastasis

**Figure 2:**
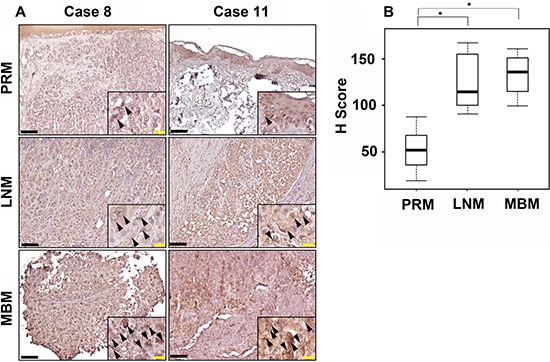
CCR4 expression during melanoma progression to brain metastasis (**A**) Representative IHC staining with anti-CCR4 antibody for PRM, LNM and MBM specimens. Black bars indicate 100 μm. The insets show a magnification of the melanoma lesions. Black arrowheads indicate CCR4-positive melanoma cells. Yellow bars indicate 20 μm. (**B**) Box plot comparing H score for PRM, LNM and MBM. *P ≤ 0.05.

The clinical relevance of the above results was validated by examining the expression of CCR4 in paired samples of primary melanoma (PRM), lymph node metastasis (LNM), and MBM derived from melanoma patients. This cohort included 12 patients. Paired triplets (PRM; LNM; MBM) were derived from 8 patients, paired duplets (PRM-LNM) or (LNM-MBM) were derived from 3 patients. A single MBM was also analyzed. IHC staining indicated that metastatic melanomas (LNM and MBM) exhibited significantly (*p* < 0.05) higher expression of CCR4 than paired PRMs (Figure 2A–2B).

### CCR4 ligands are expressed and secreted by human brain stromal cells

We previously demonstrated that the CCR4 ligands CCL17 and CCL22 are expressed in the brain [6]. Based on these results and those described above, (Figure 1), we hypothesized that the targeted migration of CCR4-expressing melanoma cells is mediated by an interaction between CCR4 expressed by melanoma cells and CCR4 ligands expressed in the brain. In order to identify the cellular source of the CCR4 ligands in the brain, we performed qRT-PCR assays using cultures of human astrocytes, microglia and brain endothelial cells and found that all 3 types of brain cells express CCL17and CCL22. It should be noted that these cells require stress conditions (e.g. starvation medium) or activation signals (e.g. exposure to melanoma-derived supernatants – see below) to express the CCR4 ligands.

**Figure 3:**
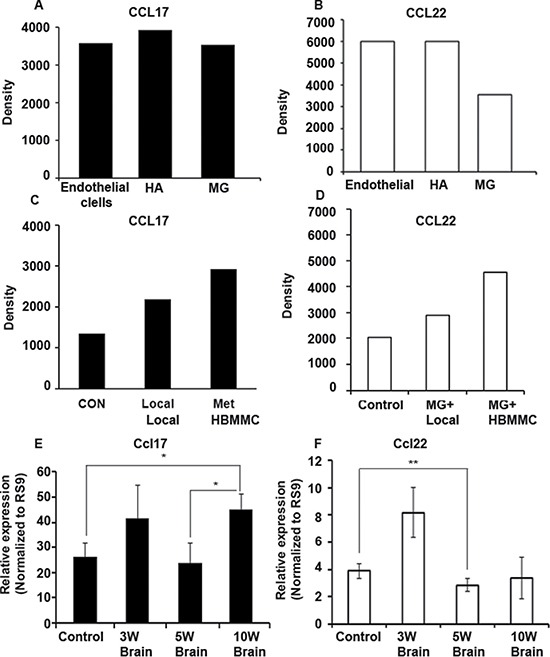
CCR4 ligands are expressed and secreted by human brain stromal cells (**A**–**B**) Chemokine secretion analysis by human chemokine array. CCL17 (A) and CCL22 (B) are secreted by human endothelial cells, astrocytes (HA) and microglia (MG). (**C**–**D**) Chemokine secretion analysis by human chemokine array. Melanoma cells alter the secretion of CCL17 (C) and CCL22 (D) by microglial cells: Microglial cells treated with local melanoma cell-conditioned media (MG+Local), treated with brain metastasizing melanoma cell-conditioned media (MG+HBMMC), microglial cells alone served as control. (**E**–**F**) The bearing of melanoma tumors leads to alterations in the expression of CCR4 ligands in the brain. (E) Nude mice were s.c inoculated with 1 × 10^6^ human brain metastasizing melanoma cells (YDFR.CB3). Three, five or ten weeks following inoculation (3W, 5W and 10W) brains were removed and analyzed for Ccl17 (E) and Ccl22 (F) expression levels using qRT-PCR. W = weeks. *p < 0.05, **p < 0.005, *n* = 3.

We next utilized a human chemokine array to evaluate secretion of the ligands from astrocytes, microglia and brain endothelial cells. These cells were incubated in starvation medium containing 0.5% FCS for 24 h. Conditioned medium collected from these cells was analyzed for the relative expression of the CCR4 ligands CCL17 and CCL22. We found that all 3 types of brain cells secreted CCL17 (Figure 3A) and CCL22 (Figure 3B, suggesting that these cells are a physiological source of the CCR4 ligands.

As shown earlier (Figure 1B) microglial cells interacted with melanoma cells and altered the expression of CCR4 in these cells. We asked whether these interactions are reciprocal, and analyzed if melanoma cells are capable of altering the secretion of CCR4 ligands from microglial cells. Using a human chemokine array (see Figure 3A–3B), we measured the secretion level of CCL17 (Figure 3C) and CCL22 (Figure 3D) from 3D cultures of microglial cells cultured with or without melanoma cells. In line with our hypothesis, microglial cells co-cultured with melanoma cells secreted higher levels of both CCL17 and CCL22 (Figure 3C and 3D) than microglial cells grown alone.

In order to test if the bearing of a subcutaneous melanoma tumor and/or the presence of melanoma micrometastasis in the brain leads to alterations in the expression of CCR4 ligands in this organ, we measured the expression of the CCR4 ligands in the brain at different time points following a subcutaneous inoculation of HBMMC to nude mice. The expression of CCR4 ligands in the brain was determined 3, 5 and 10 wks following tumor cell inoculation. The results indicated that *Ccl17* (but not *Ccl22*) expression was significantly upregulated 10 wks following tumor cell inoculation (Figure 3E–3F respectively). At this time point, no micrometastases were detected in the brain. This shows that the bearing of local, cutaneous melanoma tumors regulates the levels of CCL17 in the brain by a mechanism operating long distance. We suggest that the up-regulated levels of CCL17 in the brain could chemo-attract CCR4-expressing melanoma cells towards this organ.

### The CCR4-CCL17 axis promotes melanoma cell viability and facilitates their *in-vitro* migration

**Figure 4:**
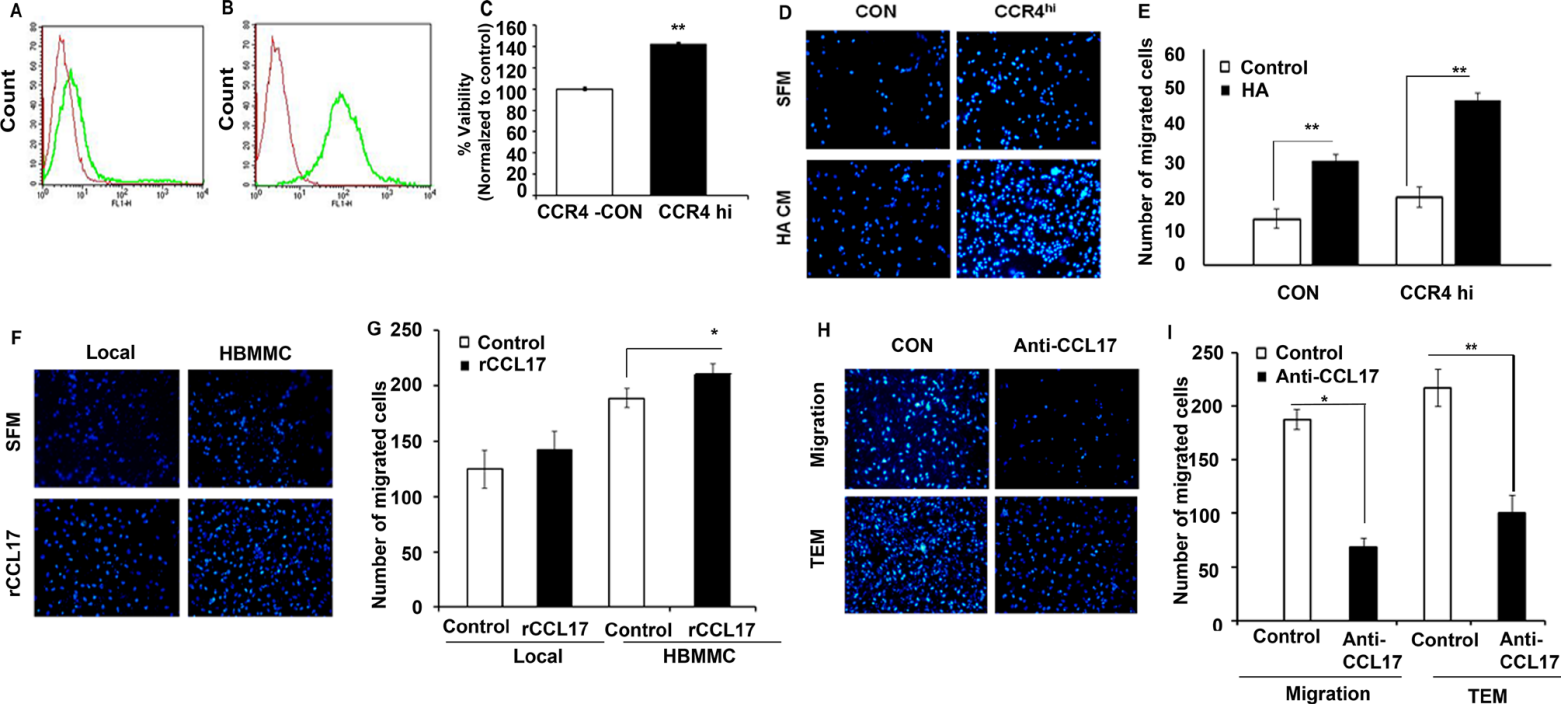
CCR4 ligands secreted by human stromal brain cells induce melanoma cell migration (**A**–**B**) FACS analysis for CCR4 expression by local melanoma cells infected with empty plasmid (CON- red) (A), or with CCR4 cDNA (CCR4^hi^-green) (B). (**C**) Viability (XTT-based assay) of control (CON) cells and of CCR4 over-expressing melanoma cells (CCR4^hi^). (**D**–**I**) Migration assays.(D) Representative images of migrated CCR4^hi^ and CON cells following 24 h incubation with astrocyte-derived soluble factors (conditioned medium - HA CM) as compared to cells that were allowed to migrate towards serum free medium (SFM). (E) Quantification of (D). (F) Representative images of migrated local and brain metastasizing melanoma cells (HBMMC) 24 h following incubation with 10 ng/ml recombinant CCL17 (rCCL17). (G) Quantification of (F). (H–I) Neutralizing anti –CCL17 Ab reduce melanoma cell migration and transendothelial migration (TEM). (I) Melanoma cells were incubated with HA CM (control) or with HA CM supplemented with 1 μg/ml neutralizing Ab to CCL17. Representative images of migration assays are shown. I Quantification of (H). Data shown are mean pixel density normalized to control (SFM). Experiments were performed in triplicate and four independent fields/well were quantified. *P < 0.05 **P < 0.005, *n* = 3.

We next asked if the CCR4-CCR4 ligand axis is involved in the viability and migratory activity of melanoma cells and in particular in their trans-endothelial migration through brain endothelial cells. To this end, we generated a CCR4-overexpressing cutaneous variant of the YDFR melanoma by viral transduction of CCR4 DNA. Control cells were transduced with scrambled CCR4 DNA (CON). These infections yielded CCR4^hi^ and CON variants, respectively (Figure 4A–4B).

To assess the effect of CCR4 on melanoma cell viability we utilized an XTT-based assay to determine the viability of CCR4^hi^ and CON cells in culture. The results (Figure 4C) show that the viability of CCR4^hi^ cells was significantly higher than that of CON cells showing that CCR4 stimulates growth of melanoma cells (Figure 4C).

Several sets of molecules including selectins and their ligands as well as chemokine receptors and their ligands are involved in trans-endothelial migration of tumor cells en route to metastatic sites [17]. We previously demonstrated that astrocytes facilitate melanoma cell migration [18]. Therefore, we asked whether the migration-enhancing effect of astrocytes is mediated, at least in part, by CCR4 ligands secreted from these cells.

The capacity of astrocyte-derived soluble factors to chemo-attract CCR4^hi^ and CON cells was assessed by utilizing transwell migration assays. Human astrocytes (HA) were added to the lower chamber of the transwell and melanoma cells (CCR4^hi^ or CON) were added to the upper well. Analysis of the results indicated that CCR4^hi^ cells migrated more efficiently in response to astrocyte-secreted soluble factors as compared with control cells (Figure 4D–4E). We next asked whether astrocyte-induced melanoma cell migration could be mediated by the CCR4 ligands CCL17 and/or CCL22. Transwell migration assays using recombinant CCL17 or CCL22 revealed that CCL17, but not CCL22 (data not shown), significantly facilitated migration of the CCR4^hi^ cells compared to control cells (CON), indicating that expression of CCR4 facilitates melanoma cell migration (Figure 4F–4G). It should be noted that the forced expression of CCR4 in melanoma cells (yielding CCR4^hi^ cells) enhanced also the spontaneous migration capacity of the melanoma cells (Figure 4D–4E).

Neutralizing CCL17 with specific Ab added to conditioned medium of the astrocytes, significantly inhibited the migration and transendothelial migration of brain metastasizing melanoma cells (Figure 4H–4I). These results show that the CCL17-CCR4 axis has a functional role in astrocyte-mediated melanoma cell migration. Thus, the interaction of CCR4, expressed by melanoma cells, with its CCL17 ligand in the brain may contributes to targeted migration of melanoma cells to the brain.

### CCR4 promotes *in-vivo* growth of cutaneous melanoma tumors and brain metastasis

**Figure 5:**
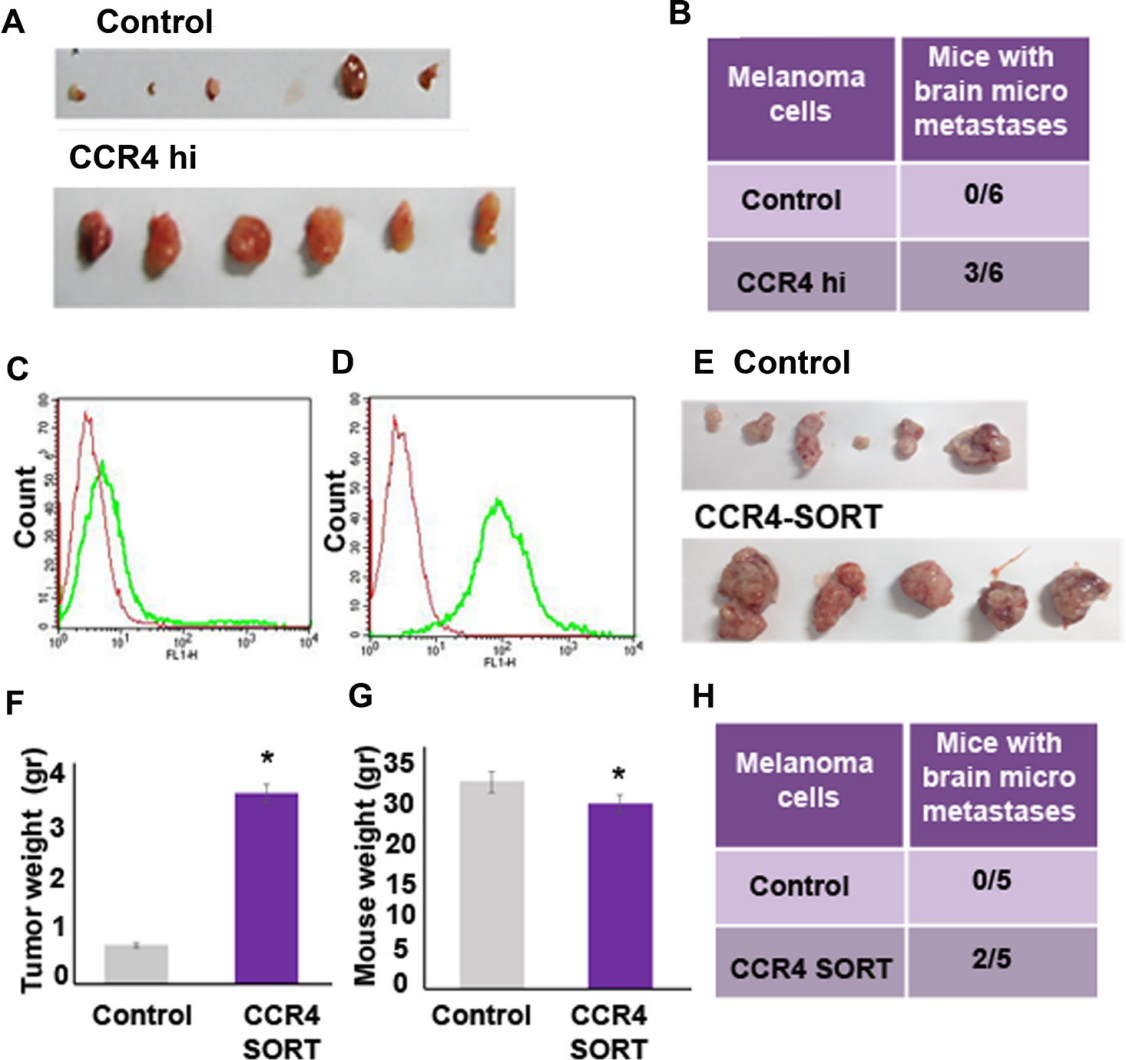
CCR4 promotes growth of cutaneous melanoma tumors and of brain metastasis The following two types of CCR4 overexpressing cells and their corresponding controls were s.c inoculated into nude mice: 1. Cells infected with CCR4 cDNA (CCR4^hi^) or control cells (empty plasmid). 2. FACS sorted cells for high CCR4 expression: Control cells (un sorted) or CCR4-sorted cells (CCR4-SORT). 12 wks following inoculation, local tumors were excised and brains were removed and analyzed for metastasis formation. (**A**) Tumors of the CCR4^hi^ and Control mouse groups. (**B**) Brain micrometastasis in the CCR4^hi^ and Control mouse groups (*n* = 6). (**C**) CCR4 expression in unsorted cells. (**D**) CCR4 expression in CCR4-sorted cells. Red-negative control, Green-CCR4. (**E**) Tumors of the CCR4-SORT and Control mouse groups. Tumor weight (gr) (**F**) and mouse weight (gr) (**G**) of mice injected with unsorted control cells or with CCR4-SORT cells. (**H**) Brain micrometastasis in the CCR4-SORT and Control mouse groups (*n* = 5). qRT-PCR was used to determine the percentage of human melanoma cells in mouse brains.

To answer the question if CCR4 plays a functional role in the growth of cutaneous melanoma and brain metastasis, CCR4^hi^ or control cells were subcutaneously (s.c) inoculated into nude mice (5 × 10^5^ cells per mouse). Local tumors were measured 3 wks following inoculation and the metastatic load in the brain was determined by a qRT-PCR based method, developed in our lab, to assess the presence of human cells in xenografted nude mice [5]. Whereas mice inoculated with control cells developed no tumors or very small ones, tumor load in mice inoculated with CCR4 over-expressing (CCR4^hi^) cells was significantly higher (Figure 5A). A quantitative evaluation of brain metastasis indicated that 50% (3 of 6) of the mice inoculated with CCR4^hi^ cells developed micrometastases in the brain, while no micrometastases were detected in the brain of mice inoculated with control cells (0 of 6) (Figure 5B).

Using flow cytometry-based cell sorting, we selected for brain metastasizing melanoma cells (HBMMC from the YDFR.CB3 variant), that endogenously express high levels of CCR4 (Figure 5C, 5D). The sorted cells, referred to here after as CCR4-SORT cells, were s.c inoculated into nude mice, as described above. The parental, unsorted HBMMC served as control (Control). Local tumors and metastatic load in the brain were measured 3 wks following inoculation. Mice inoculated with Control HBMMC developed smaller tumors than mice inoculated with CCR4-SORT HBMMC (Figure 5E, 5F). The weight of mice inoculated with CCR4-SORT was significantly lower than that of mice inoculated with Control cells suggesting that the former mice suffered from cachexia (Figure 5G). 40% (2 of 5) of the mice inoculated with CCR4-SORT cells developed brain micrometastases, while no brain micrometastases were detected in mice inoculated with Control cells (Figure 5H).

Taken together, these results clearly indicate that CCR4 is functionally involved in the tumorigenicity of melanoma as well as in the formation of brain metastasis.

### Antagonizing CCR4 ameliorates the malignancy phenotype of melanoma

To test whether pharmacological inhibition of the CCR4 axis will affect brain metastasis, we utilized the small molecule CCR4 antagonist AF-399/42016530 [19]. This antagonist blocked CCL22- and CCL17-mediated migration of human peripheral blood CD4^+^CD25^+^ Treg and Th2 cells *ex-vivo* [19] and murine Tregs *in-vivo* [20].

In the present set of experiments, we tested the effect of this CCR4 antagonist on several *in-vitro* and *in-vivo* malignancy parameters.

**Figure 6:**
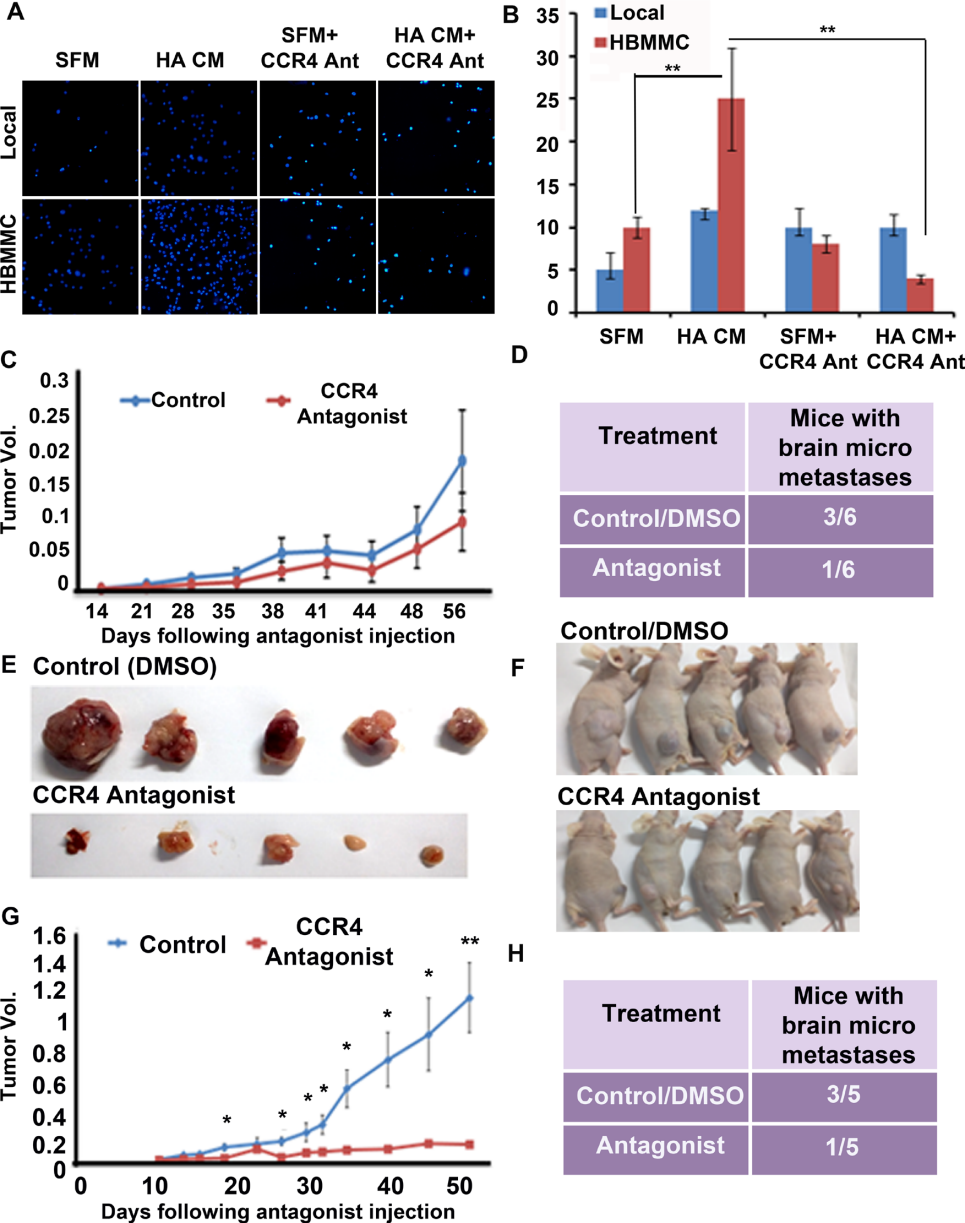
CCR4 antagonism ameliorates the malignancy phenotype of melanoma (**A**–**B**) The CCR4 antagonist reduces melanoma cell migration. Local and HBMMC were incubated in serum-free medium (SFM), SFM supplemented with migration-stimulating astrocyte-derived soluble factors (HA CM), SFM + 10 μM CCR4 antagonist, SFM supplemented with HA CM and 10 μM CCR4 antagonist. (A) Representative images of migrated local and HBMMC cells following 24 h incubation (B) Quantification of A. Data shown are number of migrated cells counted cells in four independent fields/well; **p < 0.005, ***p < 0.0005. (*n* = 3). (**C**–**D**) CCR4 over-expressing melanoma cells (CCR4^hi^) were s.c inoculated into nude mice. Two wks post cell inoculation mice were treated, twice a week, with CCR4 antagonist (1.5 μg) or with DMSO (vehicle Control). 12 wks following inoculation local tumors were excised and brains were removed and analyzed for metastasis formation. (C) Tumors of antagonist-treated and control mice. (D) Brain micrometastasis in antagonist-treated and control mice (*n* = 6). (**E**) CCR4-sorted melanoma cells were s.c inoculated into nude mice. One wk post cell inoculation mice were treated, every 2 days, with CCR4 antagonist (1.5 μg) or with DMSO (Control). 12 wks following inoculation local tumors were excised and brains were removed and analyzed for metastasis formation. (**F**) Antagonist-treated and control mice. (**G**) Tumors of antagonist-treated and control mice. (**H**) Brain micrometastasis in antagonist-treated and control mice (*n* = 5).

We demonstrated above that CCR4 facilitates melanoma cell migration towards astrocyte-derived soluble factors (Figure 4). Based on these results we evaluated the effect of the CCR4 antagonist on the migration of brain-metastasizing melanoma cells, expressing high levels of CCR4, towards astrocyte secreted factors as compared with the migration of local cutaneous melanoma cells, expressing very low levels of CCR4. Serum free medium with DMSO (the vehicle of CCR4 antagonist) served as control. The results confirmed that brain-metastasizing melanoma cells migrated better towards astrocyte-conditioned medium than local, cutaneous melanoma cells (Figure 6A–6B). The CCR4 antagonist significantly inhibited astrocyte-induced migration of brain-metastasizing melanoma cells but did not affect the migration of local melanoma cell, further supporting a role for CCR4 in facilitating brain metastasis.

In the next set of experiments, we evaluated the effect of CCR4 antagonist on tumorigenicity and metastasis formation. Nude mice inoculated s.c with CCR4^hi^ cells were treated twice weekly with 1.5 μg of CCR4 antagonist, a dose that has been optimized in previous experiments [19]. Treatment was initiated 2 wks after the inoculation of CCR4^hi^ cells. At this time point, some of the mice bear melanoma micrometastases in the brain parenchyma [18]. Control mice were treated with 10% DMSO [19]. Tumor volume was measured on a weekly basis (Figure 6C). The results (Figure 6) indicated that the CCR4 antagonist did not have a significant effect on tumor volume. The antagonist did, however influence metastasis formation. Whereas, half of the mice (3 of 6) in the control group (DMSO) developed brain micrometastases, only one of six mice (17%) in the treatment group developed micrometastases (Figure 6D).

The next *in-vivo* experiments examining the effect of triweekly treatments of the CCR4 antagonist on tumorigenesis and metastasis formation was performed with mice inoculated with HBMMC enriched for high endogenous CCR4 expression (CCR4-SORT cells). Unsorted cells served as controls.

The results indicated that the CCR4 antagonist exerted a significant effect both on local tumors as well as on metastasis (Figure 6E–6H). The tumors in the treatment group were significantly smaller than the tumors in the control (DMSO) group (Figure 6E–6G). Brain micrometastasis formation was also inhibited by the antagonist. Whereas 3 out of 5 mice (60%) in the control group developed brain micrometastases, only one mouse in the treatment group of 5 mice (20%) developed micrometastases (Figure 6H).

Taken together the results of these experiments indicated that targeting CCR4 expressed on melanoma cells ameliorates their malignant phenotype *in-vitro* and *in-vivo* suggesting that CCR4 antagonists may have a therapeutic potential in melanoma.

## DISCUSSION

The pioneering work of Muller and colleagues [21], indicated that the interactions between chemokine receptors expressed by tumor cells and the corresponding chemokine ligands expressed in various organs constitute a major mechanism for the targeted migration of these tumor cells to form site specific metastasis. This mechanism was later shown to operate in a variety of tumors using different chemokine-mediated pathways [22, 23].

CCR4 is expressed by TH2 and Treg. As such, it may serve as target in clinical situations requiring suppression of these cells [9, 24, 25]. Blockade of CCL22-CCR4 interaction by monoclonal antibodies to CCL22 significantly reduced Treg migration to ovarian tumors in experimental models [26]. CCR4 is expressed by peripheral T-cell lymphoma and T-cell leukemia and a humanized Ab directed against CCR4 is used to treat these types of cancer [27]. CCR4 is also expressed by non-lymphoid solid tumors such as breast, lung and colorectal cancer as well as by hepatocellular carcinoma. The tumor -associated CCR4 may contribute to the metastatic spread of these tumor cells [28–33]. Therefore, targeting CCR4 represents an emerging strategy for cancer therapy [34].

By employing local (cutaneous) and brain-metastasizing variants generated in nude mice xenotransplanted with cells from single human melanoma tumors (thereby ensuring a uniform genetic background of such variants), we established a molecular signature of human brain-metastasizing melanoma cells [5–7]. CCR4 was a member of this signature: Its expression was higher in cells originating in brain-metastasizing melanoma variants than in cells of the corresponding cutaneous variants [5, 6, 12]. The CCR4 ligands CCL17 and CCL22 were found to be expressed in brain [6] and their expression levels were upregulated in nude mice bearing xenografted human melanoma tumors [13]. Vemurafenib-resistant melanoma cells expressed higher levels of CCR4 than vemurafenib-sensitive cells, which are less malignant [13]. Moreover, soluble factors from the brain microenvironment upregulated CCR4 expression on melanoma cells [12].

All these findings prompted us to investigate if melanoma-associated CCR4 is functionally involved in brain metastasis. The present study shows that indeed CCR4 is a determinant of melanoma brain metastasis. This conclusion is based on several independent findings namely: Using clinical samples we validated the findings that CCR4 expression is upregulated in melanoma metastasis; ectopically upregulating CCR4 expression by melanoma cells increased their local tumorigenicity and ability to form brain metastasis; Enriching for CCR4 expression increased the malignancy phenotype of melanoma cell populations; treating melanoma-bearing mice with a small molecule CCR4 antagonist significantly reduced local tumourigenicity and metastasis formation.

Chemo attraction of CCR4-expressing melanoma cells towards CCR4 ligands in the brain seems to be a major mechanism by which this receptor exerts its pro-metastatic activity. We found that the expression of the CCR4 ligand *Ccl17* is upregulated in the brain of mice bearing local melanoma tumors but no detectable brain metastasis. This suggests that cues derived from the local cutaneous melanoma may be capable of regulating, at distance, the expression of this chemokine ligand. The increased levels of CCL17 in the brain could chemo-attract tumor cells expressing the corresponding CCR4 receptor to the brain.

In addition to the involvement of the CCL17-CCR4 axis in chemo-attracting melanoma cells to the brain, other pro-metastatic mechanisms of this axis may also be operative. For example, CCR4- expressing Tregs recruited to tumor sites may antagonize the anti- tumor activities of cytotoxic T cells thereby contributing to tumor growth. This mechanism is however not operative in xenograft models (such as the one used in this study) utilizing immune deficient mice. Another mechanism is the proliferation-boosting activity of CCR4. As demonstrated above and previously by others [35] the interaction between CCR4 expressed by tumor cells and its ligands promotes the proliferation of the tumor cells.

The results described above and elsewhere [28, 33] indicated that of the two CCR4 ligands, CCL17 is the chemokine involved in the pro-malignant functions of CCR4. CCL17 was sufficient to enhance melanoma cell invasiveness, and neutralizing this ligand in astrocyte-conditioned medium attenuated the astrocyte-mediated migration of melanoma cells.

CCL22 is apparently not involved in the migratory function of melanoma cells. Indeed a suppression of CCL22 and a concomitant increase of CCL17 levels in the cerebrospinal fluid characterize patients with highly aggressive MBM [36]. The underlying reason for the differential effect of these two chemokines on melanoma progression is not clear. Possible differences in the expression pattern of CCR4 on tumor cells vs lymphocytes might be one of the reasons as CCL22 is reported to have higher affinity for CCR4-expressing lymphocytes [37].

Based on the results of the present study we suggest that CCR4 could serve as a target for the therapy of melanoma. Clinical trials with the humanized anti-CCR4 Ab mentioned above constitute a significant step in this direction.

## MATERIALS AND METHODS

### Cell cultures

Human melanoma: The YDFR cell line was described previously [38]. The production of the cutaneous human melanoma variant (YDFR.C) and of the MBM variant (YDFR.CB3) were described previously [5], [6]. The YDFR.CB4 variant was generated as follows: 5 × 10^5^ YDFR.CB3 cells were inoculated into the left ventricle of the heart. Cells that metastasized to the brain were isolated and grown in culture to generate the fourth cycle of brain metastatic variants (YDFR.CB4). We also developed a local and human MBM variant system from the human MBM derived cell lines UCLA-SO-M12 and UCLA-SO-M16, as was described previously for the YDFR model [5, 6]. In this study, we used the metastatic variants: M12.CB2, M12.CB3 and M16.CB2 (CB2 and CB3 refer to number of cycles of re-inoculation into the left ventricle of the heart as previously described) [5].

mCherry YDFR.CB3 cells were infected to stably express mCherry (pQCXI–mCherry retroviral vector) as described previously [18] (Clontech Laboratories, Inc., USA).

CCR4^hi^ YDFR.C - YDFR.C cells were infected to over-express CCR4 (pQCXIP–mCherry retroviral vector), (CN:631516, Clontech Laboratories, Inc., USA).

Human astrocytes (HA) this cell line was purchased from ScienCell Research Laboratories, USA and maintained as described previously [18].

Human brain endothelial cells (hCMECs/D3 cells) were kindly provided by Clara Nahmias and Pierre-Olivier Couraud (Department of Cell Biology, Institute Cochin, Paris, France). These cells were used between passages 27 and 35. These cells were maintained in monolayer cultures as previously described [6, 39].

Immortalized Human Microglia - SV40, MG, cell line was purchased from ABM. The cells were maintained in monolayer cultures in Prigrow III medium (ABM, Milton, Canada).

Cells were cultured in humidified air with 6.5% CO_2_ at 37°C. The cultures were routinely tested and found to be free of mycoplasma.

### Animals

Male athymic nude-BALB/c mice were purchased from Harlan Laboratories Limited (Jerusalem, Israel). The mice were housed, maintained and used as described previously (5, 6). All experiments involving animals were approved by the TAU Institutional Animal Care and Use Committee.

### Orthotopic and intracardiac inoculation of tumor cells

Subcutaneous and Intracardiac (IC) inoculation were performed as described previously [5].

### Antibodies

Monoclonal anti-human CCR4 (R&D Systems, Minneapolis, MA, USA) was used for flow cytometry and immunofluorescence. FITC-CCR4 anti-mouse, (Jackson ImmunoResearch Laboratories, West Grove, PA, USA) was used according to manufacturer instructions.

### Flow cytometry

Human melanoma cells were cultured and FACS analyzed as previously described [6].

### Immunohistochemistry (IHC) and immunofluorescence staining

Cutaneous melanoma patients were included in the study at John Wayne Cancer Institute under the MORD-RTPCR-0995 protocol approved by the Western Institutional Review Board (WIRB Protocol #20072107). Informed consent was obtained from all subjects and the experiments performed according to the principles set out in the WMA Declaration of Helsinki and the NIH Belmont Report. Tissue specimens were de-identified and coded according to HIPAA recommendations to ensure the confidentiality of the patients.

Details are given in Supplementary materials and methods (See supplementary material).

### *In-vivo* tumorigenicity assay

To test the tumorigenic properties of derived cell lines, 1 × 10^6^ cells in 100 μl of 5% FCS RPMI-1640 medium were s.c injected into nude mice. Local SC tumors were measured once a week using a caliper as previously described [40]. Tumor volume was evaluated by the ellipsoid volume calculation formula 0.5 × (length × width^2^) [40].

### RNA preparation and reverse transcription polymerase chain reaction

Quantitative real-time polymerase chain reaction (RT-qPCR) was performed as we previously described [6]. Details are given in Supplementary materials and methods (See supplementary material).

### Chemokine secretion analysis

Human brain endothelial cells, Microglia and HAs were incubated in starvation medium for 24 h. Then cells were lysed and 100ng were analyzed for the relative expression of 36 chemokines (Proteome Profiler Human Chemokine Array Kit Panel A, R&D Systems, Canada).

### 3D matrigel culture

MBM were cultured in matrigel to form 3D culture as previously described [41]. Microglia conditioned-medium (MG CM) was prepared as previously described [18].

### Migration and transendothelial migration

Migration and transendothelial migration were performed as previously described [18].

For CCR4 antagonist-attenuated migration assays, 10 ng/ml of CCR4 antagonist (AF-399/42018025) [19, 20] were added to the upper chamber of transwell plates for 24 h stimulation.

### CCL17-induced migration and invasion assays

10 ng/ml recombinant CCL17 (R&D Systems, Canada) were added to the lower chambers of Transwell plates for 24 h of stimulation. For CCL17-blocking studies, 1 μg/ml anti-human CCL17 neutralizing Ab (R&D Systems, Canada) was added to astrocyte CM for 24 h.

### Isolation and characterization of CCR4+ sorted melanoma cells

Details are given in Supplementary materials and methods (see supplementary material).

### Treating mice with CCR4 antagonist

Mice were orthotopically inoculated with human HBMMC expressing high endogenous levels of CCR4 or (1 × 10^6^) with CCR4^hi^ cells (0.5 × 10^6^). Two wks following tumor inoculation, mice were treated with the CCR4 antagonist (SP46 - AF-399/42018025) [20], (in 10% DMSO) or with DMSO alone (control). 1.5 μg/50 μl of antagonist were injected intraperitoneally (IP) twice a week for 12 wks.

### Statistical analysis

Data were analyzed using Student's *t*-test and considered significant at *P*-values ≤ 0.05. Bar graphs represent mean and standard deviation (SD) across multiple independent experimental repeats, unless otherwise stated.

Statistical differences on the H-score values were analyzed by using the Kruskal-Wallis test among the groups. Additionally, the Steel-Dwass method was used to further compare between groups were considered statistically significant. The statistical analyses were performed with EZR (Saitama Medical Center, Jichi Medical University, Saitama, Japan, version 1.32), which is a graphical user interface for R (The R Foundation for Statistical Computing, Vienna, Austria).

## SUPPLEMENTARY MATERIALS FIGURES AND TABLES



## Abbreviations

CCR4: Chemokine (C-C motif) receptor 4
CCL17: Chemokine (C-C motif) ligand 17 (TARC)
CCL22: Chemokine (C-C motif) ligand 22 (MDC)
CLDN1: Claudin-1
Tregs: regulatory T cells
AKT: v-akt murine thymoma viral oncogene homolog 1
IC: Intracardiac
FITC: Fluorescein isothiocyanate
Ab: Antibody
HA: Human Astrocytes
hCMEC/D3: Immortalized human brain endothelial cells
RT-qPCR: Quantitative real-time PCR
IP: Intraperitoneally
wks: weeks
BRAF: v-raf murine sarcoma viral oncogene homolog B1
CCR4^hi^: CCR4 over expressing melanoma cells/CCR4 high
SFM: Serum Free Media
CM: Conditioned medium
DMSO: Dimethyl sulfoxide
ECM: Extracellular Matrix
FCS: Fetal calf serum
HBMMC: Human brain-metastasizing melanoma cells.
