# Supplementary material for: CCR4 is a determinant of melanoma brain metastasis
Source: Oncotarget. 2017 Mar 10;8(19):31079–91. doi: 10.18632/oncotarget.16076 (PMC5458190; doi:10.18632/oncotarget.16076)
Supplement: Supplementary file 1 [file oncotarget-08-31079-s001.pdf]

CCR4 is a determinant of melanoma brain metastasis

Supplementary Materials

MATERIALS AND METHODS

Immunohistochemistry (IHC)

IHC was performed on 5 µm human melanoma sections. Slides were deparaffinised, rehydrated, and then antigen retrieval was performed using 10 mM sodium citrate buffer (pH 6.0) in a boiling water bath for 20 min. The sections were then incubated in 10 g/L trichloroisocyanuric acid (Sigma-Aldrich, St. Louis, MO, USA) solution for 30 min at room temperature to bleach melanin (15). After rinse with tap water, endogenous peroxidise was blocked with hydrogen peroxide for 10 min and nonspecific binding was blocked with Protein Block Serum-Free solution (Dako, Glostrup, Denmark) for 10 min. The sections were incubated overnight with the anti-CCR4 rabbit polyclonal Ab (Bioss Antibodies Inc. Woburn, MA, USA) at a dilution of 1:100 in a 4°C humidified chamber. Visualization was performed using LSAB2 system-HRP (Dako, Glostrup, Denmark) and Liquid DAB+ Substrate Chromogen System (Dako, Glostrup, Denmark) according to the manufacturer's instructions. After visualization, the sections were counterstained with Gill's haematoxylin (Sigma-Aldrich, St. Louis, MO, USA) for 1.5 min at room temperature, dehydrated, and mounted. After the IHC, photographs were taken using a Nikon Eclipse Ti microscope and NIS elements software (Nikon, Melville, NY, USA) and analysed by ImageJ software (National Institute of Health, version 1.50i). The expression of CCR4 was quantified using H score system (16), which considers both the intensity and percentage of positive cells. The score was calculated using the formula 1 × (% of 1+ cells) + 2 × (% of 2+ cells) + 3 × (% of 3+ cells).

Immunofluorescence staining

OCT-embedded brain sections were cut. The sections were blocked with protein block (Dako, Glostrup, Denmark) for 10 min. Primary antibody (Ab), anti CCR4 (R&D Systems, Minneapolis, MA, USA); diluted 1:500, was incubated for 1 h at room temperature (RT), followed by fluorescently conjugated secondary Ab for 30 min at RT. Coverslips were mounted using Vectamount with DAPI (Vector Laboratories). The images were viewed with a × 63/1.4 oil objective, using a Leica SP5 microscope and Leica SP5 software (LAS-AF, Leica, Germany) or a confocal microscope (LSM 510, Carl Zeiss, Germany) and LSM image browser.

RNA preparation and reverse transcription polymerase chain reaction

Total RNA was extracted using the EZ-RNA Total RNA Isolation Kit (Biological Industries, Kibbutz Beit-Haemek, Israel), followed by cDNA synthesis with M-MLV Reverse Transcriptase (Ambion Inc., Foster City, CA, USA). The detection of human melanoma cells was performed as we previously described (6).

Real-time PCR reactions were run in triplicate using primers specific for human *β2M*, mouse *β2M*, human *CCL22* and human *CCL17* cDNAs. qRT–PCR was conducted with iTaq Universal SYBR Green Supermix (BioRad, CA, USA).

Isolation and characterization of CCR4+ sorted melanoma cells

Brain metastatic melanoma variant cells, YDFR. CB3, were incubated with an anti-CCR4 mAb (mouse IgG1) or a control mAb at 4°C for 30 min. After washing, cells were stained with FITC-conjugated anti-mouse IgG (Dako). Then, cells were analysed on a FACSAria (Becton Dickinson, San Jose, CA) and were sorted for isolation of CCR4 positive melanoma cell population. CCR4 positive population of melanoma cells (CCR4-SORT) were expanded as polyclonal lines for 9–14 days.
